# Developmental differences in threat learning are associated with changes in frontal-central theta activity

**DOI:** 10.1007/s00787-025-02745-2

**Published:** 2025-05-27

**Authors:** Gil Shner-Livne, Nadav Barak, Ido Shitrit, Rany Abend, Tomer Shechner

**Affiliations:** 1 School of Psychological Sciences and the Integrated Brain and Behavior Research Center, University of Haifa, Abba Hushi 199, Mt Carmel, Haifa, Israel; 2 Baruch Ivcher School of Psychology, Reichman University, Herzliya, Israel

**Keywords:** Threat learning, Adolescence, Anxiety, Theta, Alpha

## Abstract

Major theories link variations in threat learning to the emergence of anxiety symptoms, especially during adolescence. Despite significant neural maturation of threat learning circuitry during this developmental stage, research on adolescence-specific neural responses during threat learning is limited. This study was the first to examine threat learning mechanisms through neural frequency activity among youth, focusing on activity in the theta (4–7 Hz) and alpha (8–12 Hz) frequency bands. Sixty-three adolescents and 65 adults completed a two-day threat acquisition and extinction procedure, while brain activity was measured using electroencephalography (EEG). Analyses focused on developmental differences in high temporal resolution changes in frequency activity, distinguishing between induced and evoked EEG signals. Developmental differences were mainly observed in frontal-central theta activity. During acquisition, adolescents showed higher induced theta activity to both threat and safety cues than adults. During extinction, adults showed decreased evoked theta activity to the conditioned stimulus, indicating effective inhibition of threat representations; in contrast, adolescents exhibited persistent threat contingencies throughout extinction. Both groups showed consistent alpha suppression towards the threat cue, suggesting sustained vigilance even during late extinction. Frontal-central theta activity is a valuable marker for capturing differences between adolescents and adults in threat learning. These findings add to mechanistic research efforts aiming to uncover factors contributing to anxiety vulnerability during adolescence.

**Supplementary Information:**

The online version contains supplementary material available at 10.1007/s00787-025-02745-2.

## Introduction

Threat learning encompasses processes subserved by conserved neural circuitry through which neutral cues acquire or extinguish threat values. Major theories attribute a key role to aberrant threat learning processes in the emergence and maintenance of anxiety disorders [1, 2]. Developmental theories further implicate the maturation of neural pathways related to threat learning during adolescence in the increased vulnerability to anxiety symptoms during this critical period [3–5], making the mechanistic understanding of adolescence-related perturbations in threat learning, particularly at the neural level, of theoretical and clinical importance. While prior studies have identified unique patterns of threat learning during this stage, they have primarily relied on subjective reports and peripheral physiological measures [6, 7], with limited direct investigation of neural signals [8–10]. Moreover, to the best of our knowledge, no study has examined developmental differences in the frequency domain of the electroencephalography (EEG) signal during threat learning. Identifying age-related changes in oscillatory activity may shed novel light on the integrated function of the distributed neural circuitry underlying threat learning. We used event-related EEG time-frequency analysis to test whether adolescence, relative to adulthood, is associated with unique patterns of neural responses to conditioned cues during threat acquisition and extinction.

The capacity to flexibly link environmental cues to potential threats is strongly conserved across evolution, reflecting the adaptive value of threat learning processes. Such threat learning processes reflect the integrated function of a distributed neural circuitry [11, 12]. Developmental theories of anxiety posit that due to the significant adolescence-related malleability of key aspects in this circuitry, such as the links between the amygdala and the ventromedial prefrontal cortex (vmPFC) [3, 5], adolescents may be prone to maintaining fear memories, increasing their risk for developing pathological anxiety [4]. Indeed, structural and functional magnetic resonance imaging (fMRI) reveals links among developmental variations in threat learning circuitry, age-related alterations in threat learning, and anxiety [13–15].

fMRI offers high spatial resolution of brain function and therefore can provide essential insight into neural function subserving responses to threat cues [11, 12]. However, threat responses at the neural level are often rapid [16] and may not be fully captured with this methodology. EEG features high temporal resolution and thus complements fMRI by offering insights into time-dependent neural processes of interest with greater sensitivity. This methodological advantage is of particular importance when such processes reflect integrated function across distributed circuits [17], since the high-resolution EEG signal can be divided into distinct oscillatory frequency bands that correspond to synchronized brain functions. Developmental differences in brain oscillations elicited during threat learning may yield insights that complement and extend what is captured by other methods, such as event-related potentials (ERPs) or fMRI.

EEG rhythms across several frequency bands, including theta [18–22] and alpha frequency activity [23–25], reflect sensory and cognitive processes of potential relevance to threat learning [26, 27]. Studies in both rodents and humans suggest theta frequency oscillations (4–7 Hz) may correspond to connectivity between amygdala and anterior midcingulate cortex (AMC) and among other fear-related nodes [22, 28, 29] and may play a central role in fear expression [30]. For example, studies in humans have shown stronger theta activity to threat than safety cues during fear recall and extinction recall [19–21]. In a study combining EEG and fMRI, theta activity to threat cues was associated with increased activation in the amygdala during threat recall [21]. Similar to emotional and physiological responding, threat differentiation in theta activity tends to diminish after extinction learning, suggesting theta oscillations may reflect the neural representations of conditioned threat values [19–21].

Alpha oscillations (8–12 Hz) have been linked to visual attentional processes during threat anticipation, with alpha activity suppression (i.e., reduction in alpha activity relative to baseline) noted when attention is focused externally [31, 32]. Extensive evidence supports the role of visual attention in threat detection [33], with increased attentional allocation to conditioned threat stimuli demonstrated through ERPs and steady-state evoked fields [34, 35]. To date, two studies have investigated visual processing-related alpha suppression (at occipital sites) using time-frequency analysis during acquisition and extinction [23] and during a recall test one day after extinction [24]. Both studies found greater alpha suppression in response to threat than to safety cues, and this persisted even after extinction. An additional study focusing on alpha activity localized in the motor and somatosensory cortices suggested alpha suppression in response to potential-threat cues indicates preparation for aversive outcomes and necessary action [36]. Together, these findings suggest alpha suppression in visual processing areas reflects sustained vigilance towards conditioned threats, even after extinction, while alpha suppression localized in motor and somatosensory areas indicates anticipation of threat-related stimuli.

To date, only two prior EEG studies have examined developmental differences in classical threat learning, and both focused on ERP components. During acquisition, adolescents exhibited greater neural reactivity than adults, reflected in enhanced late positive potential (LPP), an event-related component reflecting the overall emotional significance of stimuli, and early ERP components [10, 37]. During extinction, adolescents showed blunted extinction responses, evidenced by enhanced differential LPP [10] and early perceptual components [37]. These developmental differences may potentially reflect significant age-dependent maturational changes in the neural circuitry subserving threat learning and regulation during adolescence [5].

Aside from these findings, developmental changes in neural responses using EEG during threat learning remain largely unexplored. Additionally, EEG frequency-domain analyses, although widely used in other areas of developmental cognitive and affective neuroscience, have not been applied to classical threat learning paradigms. To address this gap, we examined developmental differences in conditioned threat responses emerging in the frequency domain of the EEG signal.

Event-related oscillatory activity in the theta and alpha frequency bands can be divided into either an ‘evoked’ (phase-locked to a stimulus/event) or an ‘induced’ (non-phase-locked to a stimulus/event) rhythmicity [27, 38]. These spectral patterns may reflect complementary aspects of the EEG signal, occurring simultaneously and interacting with each other [39, 40]. While the functional distinction between induced and evoked signal components is not sufficiently understood, emerging data suggest they may differentially index aspects of cognitive processing [41, 42]. To date, only two studies have examined induced and evoked activity in the context of threat learning. One focused exclusively on induced alpha activity using time-frequency analysis [23], demonstrating more alpha suppression towards threat than to safety cues during threat acquisition. The other compared induced and evoked theta frequency activity in signals originating from the anterior cingulate cortex (ACC) during threat and extinction recall and found greater theta activity towards the threat cue in induced but not evoked activity [19]. Although EEG frequency activity has shown promise as a valuable marker of developmental differences in various aspects of cognitive functioning [43–45], and quantifying frequency activity appears to index distinct aspects of threat responding, no previous research has examined developmental differences in any form of frequency activity during threat learning.

To address this gap and in light of developmental theories of anxiety- and adolescence-specific perturbations in threat learning, we compared the induced (non-phase-locked) and evoked (phase-locked) theta and alpha frequency activity of adolescents and adults during threat acquisition and extinction. This was a secondary analysis of a previous study [10] that focused on the LPP [46], where we found that unlike adults, adolescents demonstrated persistent threat contingencies throughout extinction. In the present study, we extended the prior work utilizing novel time-frequency analysis.

As this was the first study to examine developmental differences in frequency EEG, we analyzed both theta and alpha activity, as they are likely involved in different aspects of threat learning, such as vigilance towards threats (alpha) and fear expression (theta). Given prior work showing comparable age-related acquisition across different modalities [6, 10, 37], we hypothesized adolescents and adults would exhibit similar differential threat acquisition, as reflected in theta and alpha frequency bands. In line with previous research showing persistent differential threat response during extinction among adolescents [10, 37], we also hypothesized adolescents, but not adults, would demonstrate similar persistent threat responses during extinction, manifesting in theta and alpha frequency bands.

## Method

### Participants

This two-visit study involved 128 participants, 63 adolescents (*M* = 15.03 years, *SD* = 1.65, Range: 12.19–17.91 years. 57.1% females) and 65 adults (*M* = 24.83 years, *SD* = 3.45, Range: 18.08–34.62 years, 55.4% females). No significant differences were found between adolescents and adults in anxiety levels, gender distribution, sleep quality, or sleep duration between the two lab visits (all *p*s ≥ 0.075) [10]. EEG data from 101 participants (47 adolescents, 54 adults) were included for threat acquisition and from 93 participants (47 adolescents, 46 adults) for extinction; see details in the Supplemental Materials. Participants were recruited through the university experiment management system (Sona) and online advertisements on social media. Adolescents (ages 12–17) were recruited via advertisements aimed at their parents, while adults (ages 18–35) were primarily recruited through Sona. All participants received modest compensation for their participation. For full details on the exclusion criteria, see the Supplemental Materials.

ERP data were reported in a previous publication [10]; all analyses reported here are new, focusing on time–frequency analytical methods to quantify theta- and alpha-band EEG activity during threat learning.

### Procedure

The study consisted of two lab visits. In visit 1, participants completed differential threat acquisition using a modified version of the bell conditioning task [6, 9, 47], adapted to include a sufficient number of trials for the EEG analysis, as required in both the original study [10] and the present one. Blue and yellow animated bells served as conditioned stimuli (CSs), counterbalanced across participants as CS+ (threat) and CS- (safety). Participants underwent three consecutive blocks of threat acquisition; each block comprised 10 presentations of the CS + and 10 presentations of the CS- (pseudorandom order). The CS + was paired with the unconditioned stimulus (UCS), a one-second loud alarm sound (95dB), in 60% of trials.

After 24 h, participants returned for a second lab visit and completed six blocks of threat extinction, followed by several self-report questionnaires. Because extinction learning tends to occur more slowly than threat acquisition, the extinction phase was designed to be twice as long as the acquisition phase [48]. Before the extinction phase, a single reminder of the CS + paired with the US and a single CS- (no US) were presented. Each extinction block consisted of 10 presentations each of the CS + and CS-. All trials during acquisition and extinction were separated by an inter-trial interval (ITI), varying randomly between 10 and 12 s. Subjective fear was assessed for each CS following each acquisition and extinction block (see below).

All experimental phases included EEG, skin conductance response (SCR), and electrocardiogram (ECG) measurements.

### Ethical considerations

Before the experiment, adult participants and the legal guardians of adolescents provided written consent, while adolescents provided written assent. Participants were informed that they could withdraw from the study at any time without any penalty. They were also informed that they would be exposed to an unpleasant but non-dangerous loud noise multiple times during the experiment. Participants were monitored by a video camera during the experiment and were able to talk with the experimenter in the adjacent room using an intercom.

All collected data were anonymized by assigning unique identification numbers, with no personal details linked to participant identities. Consent forms, which included participants’ full names, were stored separately from the experimental data in a locked cabinet within a secured room.

All procedures received ethical approval from the University’s Institutional Review Board (316/21).

### Measures

#### Self-report measures

##### Subjective fear ratings

Fear levels (‘How afraid are you when this bell appears?’) were assessed for each CS separately, prior to acquisition (pre-acquisition), following each acquisition block (3 blocks), before extinction (pre-extinction) and following each extinction block (6 blocks) using a Likert scale ranging from 1 (not at all) to 10 (extremely afraid).

##### Screen for child anxiety related emotional disorders (SCARED)

The SCARED is a commonly-used, valid, and reliable questionnaire to assess anxiety symptoms in youth [49]. It consists of 41 items rated from 0 (not true or hardly ever true) to 2 (very true or often true), for a total anxiety score ranging from 0 to 82. Internal consistency of the anxiety score in our sample was α = 0.907. Raw anxiety scores were converted into Z-scores for comparability with the adult sample [8].

##### Screen for adult anxiety related disorders (SCAARED)

The SCAARED is a valid and reliable adaptation of the SCARED assessing anxiety symptoms in adults [50]. The questionnaire consists of 44 items rated on a 3-point Likert scale, with the total anxiety score ranging from 0 to 88. Internal consistency in our study was α = 0.939. Raw scores were converted into Z-scores for comparability with the adolescent sample.

#### SCR

##### Pre-processing and analysis

Using MindWare analysis software (Version 3.0.25, OH, USA), we computed SCR as the trough-to-peak amplitude within a window between 500 ms and 7 s following CS onset. Amplitudes below 0.01µS, including negative values, were counted as zero. A. SCR scores were square-root transformed [51] and averaged per stimulus type (CS+, CS−), experimental block, and phase (acquisition, extinction). See the Supplemental Materials for recording details.

#### EEG

##### Pre-processing and analysis

All EEG data processing and analyses were performed in MATLAB (v. R2022a) using the toolboxes EEGLAB version 2022.1 [52] and FieldTrip version 20,221,212 [53]. For detailed information on recording and pre-processing, see the Supplemental Materials. All time-frequency analysis stages for induced and evoked signals in alpha and theta bands were conducted similarly and are detailed in the Supplemental Materials. Briefly stated, frequency representation of the EEG data was obtained through convolution in the time domain using Morlet wavelets ranging from 2 to15 Hz (steps of 0.5 Hz) and a Gaussian taper, with analysis windows centered every 20 ms, using 5-cycle wavelets (Morlet constant of m = 5) [23]. Frequency activity was baseline-corrected to a window between 700 ms and 200 ms before stimulus onset [23] and normalized to pre-stimulus power on a dB scale. Full details are in the Supplemental Materials.

For statistical analysis of theta-band activity (4–7 Hz), we used the mean power at frontal-central electrodes (Fz, FC1, FC2) within 200–600 ms after CS onset, based on visual inspection. For alpha-band activity, we used the mean power from 8 to 12 Hz at the occipital-parietal electrode site (Oz, Pz, PO3, PO4), within 500–1200 ms after CS onset [23, 24]. To enhance the reliability of frequency activity indices while maintaining temporal order during extinction learning, we averaged all three acquisition blocks, the first three blocks of extinction (reflecting ‘early extinction’), and the last three blocks of extinction (reflecting ‘late extinction’). We aimed to provide enough trials for a robust EEG signal analysis while maintaining consistency across phases [54]. Full results of the total (evoked + induced) EEG signal are presented in the Supplemental Materials.

### Statistical analysis

Our primary analyses focused on developmental differences in differential threat acquisition and extinction in evoked and induced theta and alpha activity and their moderation by anxiety. For acquisition, we used repeated-measures analyses of covariance (RM-ANCOVAs) of theta and alpha activity, in both induced and evoked signal aspects. Stimulus (CS+, CS) was the within-subject factor, and Age group (adolescents, adults) was a between-subjects factor, while Anxiety levels (Z-scores of SCARED/SCAARED) were entered as a continuous covariate. Similar analysis was used for extinction, with Phase (early extinction, late extinction) as an additional within-subject factor. Greenhouse-Geisser or Huynh-Feldt corrections were applied as necessary. Significance was set at *p* <.05 (two-tailed), and Bonferroni corrections were applied for multiple comparisons. All statistical analyses were conducted using SPSS (version 27).

## Results

Results of subjective self-report, SCR, and LPP measures during acquisition and extinction are reported in full in Shner-Livne et al. [41]. In short, both adolescents and adults demonstrated successful differential threat acquisition indexed by self-reported fear, SCR, and LPP. Compared to adults, adolescents showed heightened SCR and LPP responses to both threat and safety cues and exhibited stronger threat-specific SCR. During extinction, both adolescents and adults showed comparable learning as indicated by self-reported fear and SCR, but only adolescents displayed persistent differential LPP responses.

We present the results for theta and alpha frequency bands across induced and evoked signal components separately for threat acquisition and extinction. To facilitate readability, we only report significant results for each RM-ANCOVA model. We use time-frequency representations and topographical figures to depict the frequency activity. Time-frequency representations reflect changes in frequency activity over time in specific channels after the CS + and CS- presentations. Topographic representations display the distribution of frequency activity across the scalp in specific frequency bands following the CS + and CS- presentations within the statistical analysis time window.

### Threat acquisition (Visit 1)

#### Theta frequency band

##### Induced activity

**Fig. 1:**
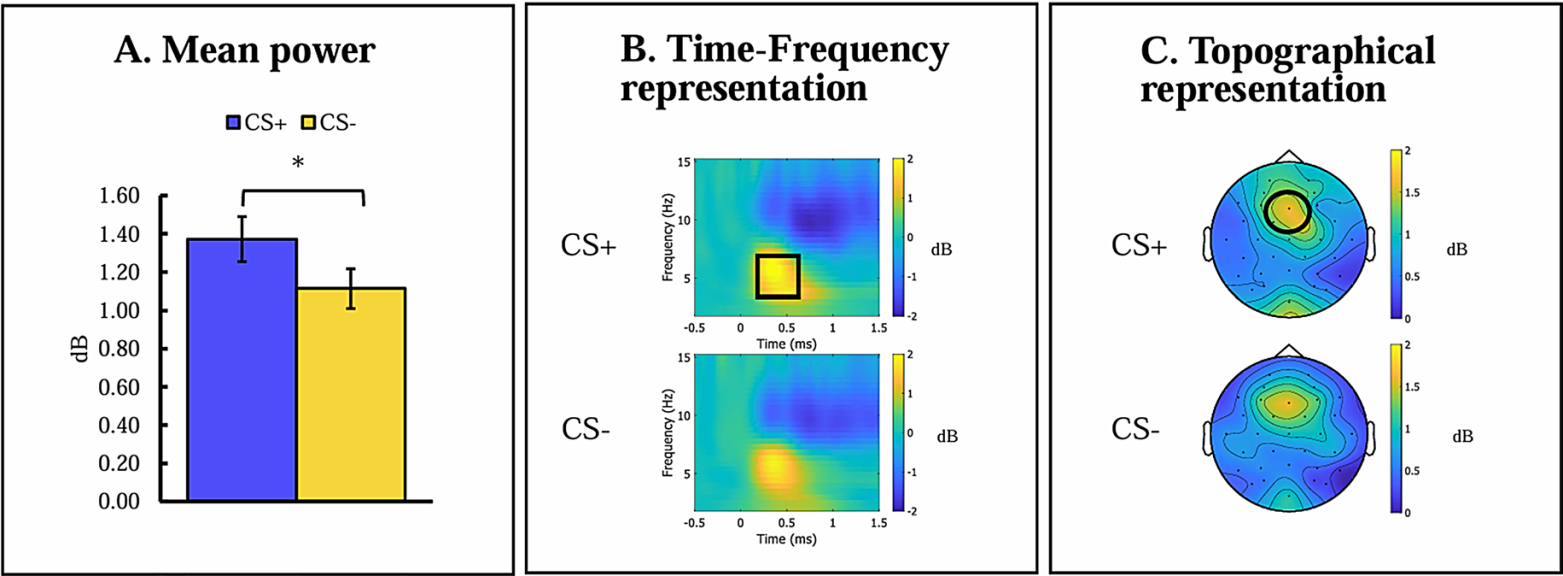
Induced theta frequency band during threat acquisition. (**A**) Mean power of induced theta activity at frontocentral sites (Fz, FC1, FC2) within 200-600 ms post-stimulus onset. (**B**) Time frequency representation of induced theta activity at frontocentral sites (Fz, FC1, FC2) during CS+ and CS- trials. (**C**) Topographic presentation of frequency activity 200-600 ms post-stimulus onset during CS+ and CS- trials. Notes: Error bars represent the standard error of the mean; black squares represent the time window used for all statistical analysis; black circles represent the electrodes used for all statistical analyses; CS+, conditioned threat cue; CS-, conditioned safety cue; dB, band-power after decibel conversion. * *p* <.05

RM-ANCOVA testing the Stimulus (CS+, CS-) x Age (adolescents, adults) x Anxiety effect on induced theta activity yielded a main effect of Stimulus, *F*(1,96) = 4.163, *p* =.044, *η*_*p*_^*2*^ = 0.042. Participants exhibited greater theta activity to the CS+ (*M* = 1.384, *SD* = 1.184) than to the CS- (*M* = 1.121, *SD* = 1.024), suggesting successful acquisition of conditioned threat response. A main effect of Age emerged, *F*(1,96) = 4.641, *p* =.034, *η*_*p*_^*2*^ = 0.046, indicating greater overall theta activity among adolescents (*M* = 1.447,*SD* = 0.895) than adults (*M* = 1.057,*SD* = 0.895). No other significant effects were observed. Statistical differences, time-frequency, and topographic representations of induced theta activity during acquisition are presented in Fig. 1.

##### Evoked activity

RM-ANCOVA with Stimulus (CS+, CS-) x Age (adolescents, adults) x Anxiety did not yield any significant effects (all *p*s ≥ 0.277).

Statistical differences, time-frequency, and topographic representations of theta activity during acquisition are presented in Fig. 1. For a full and comhrehensive comparison of induced and evoked theta results, see Figure S1 in the Supplemental Materials.

#### Alpha frequency band

##### Induced activity

**Fig. 2:**
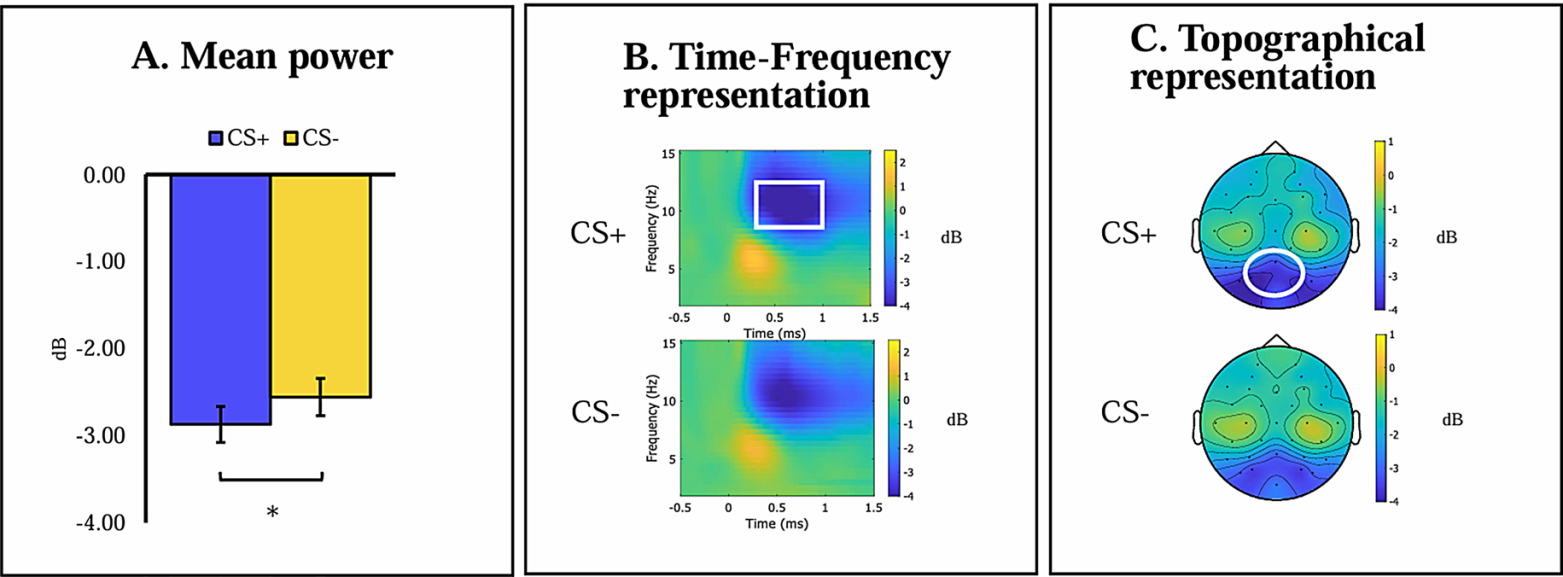
Induced alpha frequency band activity during threat acquisition. (**A**) Mean power of induced alpha activity at occipito-parietal sites (Oz, Pz, PO3, PO4) in a time window of 500-1200 ms post-stimulus onset. (**B**) Time frequency representation of induced alpha activity at occipito-parietal sites (Oz, Pz, PO3, PO4), during CS+ and CS- trials. (**C**) Topographic presentation of frequency activity 500-1200 ms post-stimulus onset during CS+ and CS- trials. Notes: Error bars represent the standard error of the mean; white squares represent the time window used for all statistical analysis; white circles represent the electrodes used for all statistical analyses; CS+, conditioned threat cue; CS-, conditioned safety cue; dB, band-power after decibel conversion. * *p* <.05

RM-ANCOVA with Stimulus (CS+, CS-) x Age (adolescents, adults) x Anxiety yielded a main effect of Stimulus, *F*(1,96) = 6.889, *p* =.010, *η*_*p*_^*2*^ = 0.067. Participants exhibited greater alpha suppression to the CS+ (*M*=-2.874, *SD* = 2.079) than the CS- (*M*=-2.554, *SD* = 2.179), suggesting successful differential threat response indexed by induced alpha activity. No other significant effects were observed. Statistical differences, time-frequency, and topographic representations of induced alpha activity during acquisition are presented in Fig. 2.

##### Evoked activity

RM-ANCOVA with Stimulus (CS+, CS-) x Age (adolescents, adults) and Anxiety levels as a covariate did not yield any significant effects (all *p*s ≥ 0.079).

For a full and comhrehensive comparison of this induced and evoked theta results, see Figure S2 in the Supplemental Materials. To summarize, both adolescents and adults exhibited successful threat acquisition, as reflected in induced theta and alpha activity. However, developmental differences were observed specifically in induced theta activity, with adolescents showing heightened responses to both threat and safety cues. This pattern may suggest greater but less specific neural sensitivity in the context of potential threat, and extends prior work showing similar results in LPP and magnetoencephalogram (MEG) measures [10, 55].

### Threat extinction (Visit 2)

#### Theta frequency band

##### Induced activity

RM-ANCOVA with Stimulus (CS+,CS-) x Phase (early, late) x Age (adolescents, adults) x Anxiety yielded a main effect of Phase, *F*(1,90) = 9.443, *p* =.003, *η*_*p*_^*2*^ = 0.095, suggesting a decrease in overall theta activity between the early (*M* = 1.02, *SD* = 0.80) and late (*M* = 0.728, *SD* = 0.90) phases of extinction. No other significant effects were observed.

##### Evoked activity

**Fig. 3:**
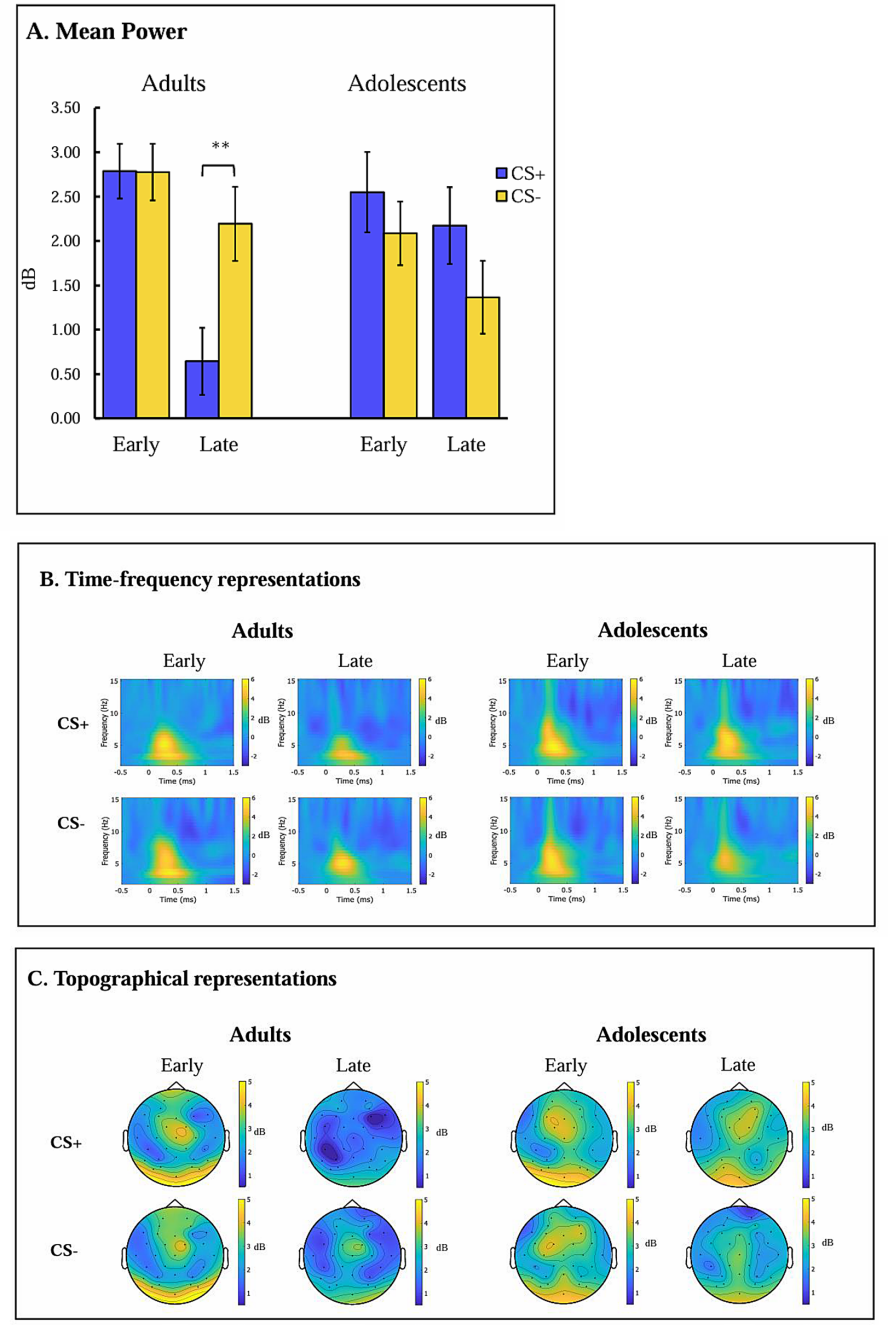
Evoked Theta frequency band during threat extinction. (**A**) Mean power of evoked activity among both adults and adolescents at frontocentral site (Fz, FC1, FC2) in a time window of 200–600 ms post-stimulus onset. (**B**) Time frequency representation of evoked activity among adults and adolescents at frontocentral sites (Fz, FC1, FC2), during CS + and CS- trials in early and late phases of extinction. (**C**) Topographic presentation of evoked activity among adults and adolescents 200–600 ms post-stimulus onset, during CS + and CS- trials in early and late phases of extinction. Notes: Error bars represent the standard error of the mean; CS+, conditioned threat cue; CS-, conditioned safety cue; dB, band-power after decibel conversion. * *p* <.05; ** *p* <.01

RM-ANCOVA with Stimulus (CS+,CS-) x Phase (early extinction, late extinction) x Age (adolescents, adults) x Anxiety yielded a significant three-way interaction of Stimulus x Phase x Age, *F*(1,89) = 4.558, *p* =.036, *η*_*p*_^*2*^ = 0.049. Follow-up RM-ANOVA with Stimulus (CS+,CS-) x Phase (early extinction, late extinction) for each age group revealed a significant Stimulus x Phase interaction only among adults, *F*(1,44) = 5.755, *p* =.021, *ηp2* = 0.116. Follow-up analyses revealed that while adults’ responses to the CS + decreased from early to late extinction, *t*(44) = 5.140, *p* <.001, their responses to the CS- did not change across extinction phases, *t*(44) = 1.256, *p* =.216. The changes during the late extinction phase resulted in higher evoked theta activity towards the CS- (*M* = 2.193, *SD* = 2.537) than the CS+ (*M* = 0.644, *SD* = 2.538), *t*(44)=-3.592, *p* =.001. In contrast, adolescents exhibited a main effect of stimulus only at a trend level, *F*(1,46) = 3.535, *p* =.066, *ηp2* = 0.071, suggesting consistent higher response to the CS + than the CS- across extinction. Statistical differences, time-frequency, and topographic representations of evoked theta activity during extinction are presented in Fig. 3. For a full and comhrehensive comparison of the induced and evoked theta results, see Figure S3 in the Supplemental Materials.

#### Alpha frequency band

**Fig. 4:**
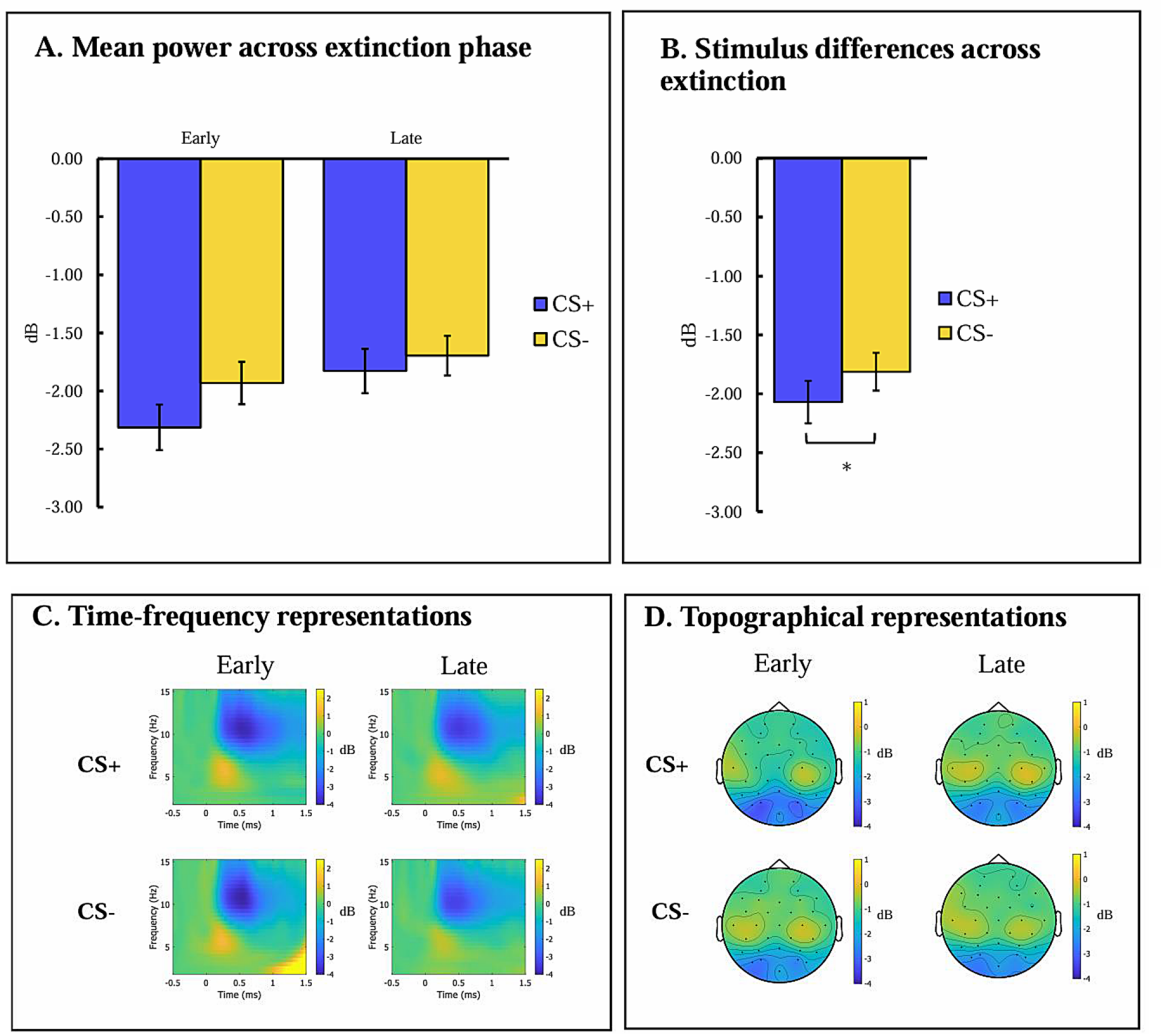
Induced alpha frequency band during threat extinction. (**A**) Mean power of induced activity among all participants at occipito-parietal sites (Oz, Pz, PO3, PO4) in a time window of 500-1200 ms post-stimulus onset. (**B**) Induced activity during CS+ and CS- across all extinction trials. (**C**) Time frequency representation of induced alpha activity among all participants at occipito-parietal sites (Oz, Pz, PO3, PO4) during CS+ and CS- trials in early and late phases of extinction. (**D**) Topographic presentation of induced alpha activity among all participants 500-1200 ms post-stimulus onset during CS+ and CS- trials in early and late phases of extinction. Notes: Error bars represent the standard error of the mean; CS+, conditioned threat cue; CS-, conditioned safety cue; dB, band-power after decibel conversion

Statistical differences, time-frequency, and topographic representations of alpha activity during extinction are presented in Fig. 4.

##### Induced activity

RM-ANCOVA with Stimulus (CS+, CS-) x Phase (early extinction, late extinction) x Age (adults, adolescents) x Anxiety yielded a main effect of stimulus, *F*(1,90) = 6.877, *p* =.010, *ηp2* = 0.071, suggesting greater alpha suppression to the CS+ (*M*=-2.070,*SD* = 1.736) than the CS- (*M*=-1.812,S*D* = 1.553). A main effect of phase emerged, *F*(1,90) = 9.245, *p* =.003, *ηp2* = 0.093, suggesting a decrease in induced alpha suppression between the early (*M*=-2.12,*SD* = 1.72) and late (*M*=-1.76,*SD* = 1.65) phases of extinction. No other effects were observed.

##### Evoked activity

RM-ANCOVA with Stimulus (CS+, CS-) x Phase (early extinction, late extinction) x Age (adults, adolescents) and Anxiety as a covariate did not yield any significant effects (all *p*s ≥ 0.088). For a full and comhrehensive comparison of the induced and evoked alpha results, see Figure S4 in the Supplemental Materials.

To summarize, during extinction, developmental differences were observed specifically in evoked theta activity. While adults showed a reduction in evoked theta responses to the threat cue, adolescents maintained differentiation between threat and safety cues, suggesting reduced flexibility in adapting to changing threat contingencies. This pattern may indicate that adolescents have a diminished capacity to regulate their threat responses once the threat is no longer present, as previously observed across species [4, 56].

## Discussion

We examined developmental differences in theta (4–7 Hz) and alpha (8–12 Hz) brain frequency activity, comparing adolescents and adults during threat acquisition and extinction learning. Two major findings emerged. First, adolescents and adults demonstrated comparable differential threat acquisition in both theta and alpha bands, indicating threat learning manifests in induced theta and alpha activity already in adolescence. Second, during extinction, developmental differences were observed in evoked theta activity. These findings extend prior work by suggesting adolescence-specific difficulty extinguishing threat responses is associated with evoked frontal-central theta activity.

During the acquisition phase, adolescents and adults exhibited successful threat learning, as indicated by differential frequency activity (CS + vs. CS-) in both induced theta activity and alpha suppression. These findings are in line with previous studies in adults showing differential conditioned activity in theta [19–22, 57] and alpha bands [23–25, 57–59]. We extend these findings by demonstrating similar patterns of threat acquisition are present in adolescents, namely, pronounced induced theta and alpha suppression when responding to threat cues. To the best of our knowledge, this is the first study to demonstrate threat learning mechanisms indexed by neural frequency activity among youth.

Moreover, compared to adults, adolescents displayed an overall heightened induced theta activity during acquisition. This developmental finding is consistent with previous studies reporting enhanced early ERP components [37] and LPP in adolescents [10] compared to adults during acquisition. A MEG study also demonstrated stronger activation among adolescents during fear learning and generalization consistent with LPP timing [55]. Together, our results add to a growing literature suggesting adolescents demonstrate greater neural reactivity, alongside physiological reactivity, than adults during threat acquisition. Since these effects are not cue-specific, they may reflect hypersensitivity to potential threat context evidence across different neural indices. However, factors such as increased arousal, attentional differences, or skull thickness [60] could also influence this age-related effect and should be considered when interpreting these findings.

During extinction, developmental differences emerged in evoked theta activity. Adults demonstrated a decrease in evoked theta response to the threat cue over the course of extinction, while adolescents appeared to maintain the threat contingency throughout extinction, suggesting a lack of flexible adaptation. Evoked theta towards the safety cue remained stable during extinction, suggesting it specifically indexes threat values. This is consistent with a previous adult study integrating EEG and fMRI [21], which proposed the suppression of theta activity mirrors a parallel decreased amygdala activity and increased vmPFC activity when the threat cue no longer predicts danger. Reduction in theta activity may therefore correspond to a reduction in fear circuitry activation, as the threat-predictive value of the CS + diminishes during extinction [61]. Lack of theta activity inhibition in response to the CS + among adolescents may reflect a diminished ability to adapt to changes in potential threats and may potentially contribute to slower extinction and increased risk for developing anxiety [4, 5].

Consistent with findings from a prior study examining alpha suppression during extinction-recall [24], we observed persistent differential alpha suppression throughout the relatively long extinction phase in both age groups [24]. The persistence of threat contingencies may interfere with learning the new safety contingency during extinction and could explain why some individuals remain vigilant even when other fear responses diminish.

We examined developmental differences in frequency EEG activity during threat learning. Our analytical approach enabled us to dissociate the EEG signal into induced (non-phase-locked) and evoked (phase-locked) components in theta and alpha frequency bands. Developmental differences emerged in both induced and evoked theta activity. Adolescents showed increased overall induced theta during acquisition compared to adults, while adults but not adolescents showed a decrease in evoked theta towards the threat cue during extinction. Although the specific roles of induced and evoked activity remain to be elucidated, they may reflect age-moderated aspects of prefrontal-subcortical interactions associated with threat learning and threat regulation, in line with prior findings demonstrating developmental effects in such circuitry [40, 63, 64].

Induced theta has been linked to changes in neuronal synchronization associated with higher cognitive processes, including learning and expectation formation [62–64]. In adults, induced theta during threat processing has been suggested to be localized at the AMC [19], and may reflect functional coupling between the AMC and the amygdala [21]. The overall higher induced theta activity observed in adolescents during threat acquisition may reflect adolescence-specific heightened activity within this fear-regulatory circuit. Indeed, in our prior report, adolescents demonstrated enhanced physiological responding during acquisition relative to adults [10]. Given that induced theta is not cue-locked, heightened theta power may correspond to excessive, context-dependent preparation for aversive events [16]. Because the prefrontal-amygdala regulatory network continuously matures during adolescence [5], higher induced theta activity may index less efficient top-down control over the amygdala in such settings and contribute to adolescents’ heightened risk for developing anxiety symptomatology.

In contrast, evoked theta is comprised of stimulus-driven, phase-locked oscillations, resulting from transient excitatory and inhibitory neuronal activities [64, 65]. To the best of our knowledge, no study has directly examined evoked theta in the context of threat acquisition and extinction in humans. However, research in rodents suggest theta phase-resetting (which constitutes evoked theta activity) during threat learning involves inhibitory neurons in the dorsomedial prefrontal cortex (dmPFC), which modulate interactions between the dmPFC and the amygdala, thereby influencing sensory processing and fear regulation [66]. This phase-resetting is thought to reflect the reactivation of conditioned associations between threat cues and aversive outcomes, eliciting behavioral fear responses. Notably, this effect peaks during recall one day after acquisition but diminishes with extended extinction [66]. Based on these findings, diminished evoked theta power during late extinction may reflect successful extinction-driven inhibition of fear expression circuits observed in adults but not in adolescents.

Taken together, these findings suggest that both induced and evoked theta may correspond to distinct aspects of neural circuitry activation during threat learning which are differentially modulated by age. Specifically, heightened induced theta in adolescents may indicate less efficient top-down amygdala regulation, while differences in evoked theta during extinction suggest a developmental shift in fear regulation. These results underscore adolescence as a sensitive period for emotional processing, with implications for age-related differences in anxiety vulnerability.

The study had several limitations. First, developmental differences in frequency activity may be influenced by unassessed factors such as pubertal stage, hormone levels, or prior adversity, as well as threat-related mechanisms like threat appraisal, perceptual sensitivity, and physiological arousal. Future research should incorporate these factors to better isolate developmental effects on frequency activity. Second, the number of participants excluded from analysis because of data rejection, mainly resulting from artifacts, exceeded our initial target. The extended task duration required for concurrent EEG and SCR recordings may have posed challenges in sustaining participant engagement, potentially leading to increased movements and artifacts. Given the modest sample size, caution is needed when generalizing our findings to broader populations. Future studies with larger samples could assess whether these effects hold across diverse demographic and clinical groups. Third, due to the limited capacity (32-channels) of the EEG device used, we were unable to perform precise source localization [67]; consequently, interpretations of signal origins are conservative and rely on prior studies. Future research is encouraged to utilize source localization techniques or simultaneous EEG-fMRI recordings for a more comprehensive understanding of the neural mechanisms underlying these developmental differences in theta frequency activity. This approach could provide deeper insights into how neural oscillatory dynamics interact with activity within specific brain regions and networks across development, also enhancing our mechanistic understanding of both induced and evoked neural activity. Further, integrating methods such as non-invasive brain stimulation and EEG [62, 68] could shed light on the role of more specific regions in the observed effects and examine the effects of modulating target frequencies.

In summary, our findings highlight theta activity, both induced and evoked, as a marker for developmental differences during threat acquisition and extinction. These results make a novel contribution to the growing mechanistic literature on developmental variations in threat learning and suggest that frequency analysis could be a valuable method for identifying neurobiological markers associated with heightened threat responses in adolescents, thus helping to identify increased risk for developing anxiety. While future research is needed to replicate and extend these findings in developmental and clinical populations, a recent study [9] has linked extinction impairments, measured via EEG, to poorer therapeutic outcomes. Building on this, a deeper understanding of these neural mechanisms may eventually contribute to the refinement of personalized anxiety interventions, for example, with the optimization of exposure therapy strategies [69, 70]. Additionally, theta oscillations are implicated in the neural circuitry underlying threat learning; thus, understanding these mechanisms could inform non-invasive neuromodulation techniques targeting fear and anxiety circuits [71, 72]. Such techniques, including transcranial magnetic stimulation (TMS) and transcranial direct current stimulation (tDCS), could be incorporated as adjuncts to exposure therapy, potentially modulating the inhibition of threat responses during extinction or exposure sessions.

## Electronic supplementary material

Below is the link to the electronic supplementary material.

Supplementary Material 1

## Data Availability

The data and codes that support the findings of this study are available from the corresponding author, upon reasonable request.
