# Supplementary material for: Developmental differences in threat learning are associated with changes in frontal-central theta activity
Source: Eur Child Adolesc Psychiatry. 2025 May 27;34(11):3387–99. doi: 10.1007/s00787-025-02745-2 (PMC12647258; doi:10.1007/s00787-025-02745-2)
Supplement: Supplementary file 1 — Supplementary Material 1 [file 787_2025_2745_MOESM1_ESM.docx]

**Supplemental Materials**

**Supplemental Methods**

***Participants***

This two-visit study involved 128 participants, 65 adults (*M*=24.83 years, *SD*=3.45, Range: 18.08-34.62 years, 55.4% females) and 63 adolescents (*M*=15.03 years, *SD*=1.65, Range: 12.19-17.91 years. 57.1% females). Of these, 3 participants (1 adult and 2 adolescents) aborted during threat acquisition, and an additional 9 (5 adults and 4 adolescents) did not return to the second lab visit. Overall, 59 adults and 57 adolescents completed both experimental visits. Individuals were excluded from the study if they met any of the following criteria: current use of psychotropic medications, a history of head injuries or any current psychiatric disorder including attention deficit hyperactivity disorder, or color blindness [1]. For adult participants, exclusion criteria were assessed through self-reported declarations in the departmental experiment management system (Sona). For adolescent participants, exclusion criteria were determined through phone screening with their parents before the adolescents arrived at the lab.

***Measures***

**Skin Conductance Recording (SCR).** Skin conductance was continuously recorded at 500 Hz with an 8-slot Bionex system (Mindware Technologies Ltd., OH, USA) and measured using two isotonic gel electrodes placed on participants’ left palm (i.e., hypothenar and thenar muscles).

**Electroencephalogram (EEG).** During all experimental tasks, EEG was recorded using a g.Nautilus RESEARCH 32 g.SCARABEO (g.tec, medical engineering GmbH, Austria) wearable headset with 32 Ag/AgCl electrodes. Electrodes were placed according to the standard 10-20 international system (FP1, FP2, AF3, AF4, F7, F3, Fz, F4, F8, FC5, FC1, FC2, FC6, T7, C3, C4, Cz, T8, CP5, CP1, CP2, CP6, P7, P3, Pz, P4, P8, PO7, PO3, PO4, PO8, Oz). Two additional electrodes were used, with AFz as a ground electrode and an active sintered Ag/AgCl electrode at the right earlobe as a reference electrode. According to the manufacturer’s instructions, electrode impedances were kept below 30 kΩ before recording. Data were amplified at a 24-bit resolution and sampled at a rate of 500 Hz.

***Analytic Procedure***

**Pre-Processing.** For pre-processing, we used guidelines for time-frequency analysis [2], and developmental considerations as stated by the Maryland Analysis of the Developmental EEG (MADE) pipeline [3].

First, EEG data were high-pass filtered using a 2nd-order infinite impulse response (IIR) Butterworth filter (half-amplitude cutoff=0.1 Hz and slope=12 dB/octave). Next, EEG data were visually inspected for flat/noisy channels; these were deleted and interpolated during later stages of the pre-processing. Channels with high impedance according to the manufacturer (above 100 kΩ) during the recording were specifically inspected. According to the MADE pipeline, subjects with more than three bad channels (10% of the channels) were excluded from the EEG analysis [3]. Global noisy/bad channels were excluded from all artifact rejection and correction processes and were interpolated in later stages of analysis.

Independent component analysis (ICA) was then applied to the continuous data. ICA components were manually inspected, and those corresponding to eye movements and blinks were corrected (0-4 components for each individual). Continuous data were segmented into epochs ranging from -2500 ms to 7500 ms relative to CS onset (10 seconds total). Thereafter, an automatic artifact detection algorithm implemented in the ERPLAB toolbox was employed for artifact detection and rejection in the segmented data. Trials were excluded if the peak-to-peak voltage within the EEG epoch was greater than 300 μV in any 200 ms window in any channel. To ensure a good signal-to-noise ratio, subjects with more than 25% rejected trials across the whole task (acquisition and extinction separately) were excluded from the EEG analysis [4]. For this reason, 18 participants (4 adults and 14 adolescents) were excluded from the acquisition analysis, and 12 participants (5 adults and 7 adolescents) were excluded from the extinction analysis. Global channels with poor data quality were interpolated before average re-referencing. An additional participant was excluded from the acquisition analysis due to more than 10% globally noisy channels [3]. Finally, an additional 6 participants (adults) were excluded from acquisition and 10 (8 adults and 2 adolescents) from the extinction EEG analysis due to technical issues during the recording. For a summary of participants excluded from the EEG analysis, see Table S1.

| **Table S1.** Number of participants excluded from the final EEG analysis due to noisy data or technical issues (25%<rejected trials, and/or 10%<global bad channels) | | | | | |
| --- | --- | --- | --- | --- | --- |
| Rejection criteria |  | Adults | Adolescents | Total |  |
| 25%<rejected trials | Acquisition | 4 | 14 | 18 |  |
|  | Extinction | 5 | 7 | 12 |  |
| 10%<globally noisy channels | Acquisition | 1 | 0 | 1 |  |
|  | Extinction | 0 | 0 | 0 |  |
| Technical issues during EEG recording | Acquisition | 6 | 0 | 6 |  |
|  | Extinction | 8 | 2 | 10 |  |
| **Total rejected participants** | Acquisition | 11 | 14 | **25** |  |
|  | Extinction | 13 | 9 | **22** |  |

To conclude, 101 participants were included in the EEG analysis of the acquisition and 93 in the EEG analysis of the extinction. For a detailed summary of the number of rejected trials among participants included in the final EEG analysis, see Table S2. An independent samples t-test revealed no significant differences between adults and adolescents in the number of rejected trials included in the final analysis of the acquisition and the extinction phases, *t*(99)=-1.270, *p*=.230, *t*(91)=-.901, *p*=.370, respectively.

| **Table S2.** Percentages of rejected trials among participants included in the final EEG analysis | | | | | | |
| --- | --- | --- | --- | --- | --- | --- |
|  | Adults | | | Adolescents | | |
|  | *M* | *SD* | Range | *M* | *SD* | Range |
| Acquisition | 7.06% | 6.88 | 0-23.33 | 8.72% | 6.96 | 0-25.00 |
| Extinction | 7.41% | 6.73 | 0-23.26 | 8.65% | 6.56 | 0-24.88 |

Global channels with poor data quality (due to high impedance during recording or technical issues) were interpolated using the spherical spline method prior to the average re-referencing. Finally, all EEG channels were re-referenced to the average of all electrodes.

**Time-Frequency Analysis.** The time-frequency analyses were conducted similarly for the induced and evoked aspects of the EEG signals, in both alpha and theta frequency bands. The evoked activity was computed for each participant by averaging all trials per CS and then running a time-frequency analysis on the averaged signal (i.e., synchronized in phase). The induced activity was calculated by subtracting the individual evoked activity from the total activity in each trial of the EEG signal of each CS [5] and then running a time-frequency analysis on the remaining signal in each of the trials [6, 7].

Time-frequency analysis was performed in 10-second EEG epochs that began 2.5 seconds prior to CS onset and lasted until 7.5 seconds following CS onset in each artifact-free trial. The frequency representation of the EEG data was obtained through convolution in the time domain using Morlet wavelets ranging from 2 to 15 Hz (in steps of 0.5 Hz) and a Gaussian taper, with analysis windows centered every 20 ms, using 5-cycle wavelets (Morlet constant of m=5) [7].

The frequency and temporal smoothing (i.e., Gaussian standard deviation) were derived from the wavelet equation, based on recommendations for analysis and publication of time-frequency analysis [8]. The following equations were used:

1. $\sigma f=\frac{f_{0}}{m}$ for frequency precision
2. $\sigma t=\frac{1}{2\times\pi\times\sigma f}$ for time precision

where $f_{0}$ refers to the frequency of interest (Hz), and $m$ refers to the Morlet constant.

Wavelets with a center frequency between 8 Hz and 12 Hz were used to quantify alpha band activity. At 8 Hz, the temporal precision was 99 ms, and the frequency precision was 1.6 Hz. At 12 Hz, the temporal precision was 66 ms, and the frequency precision was 2.4 Hz. Wavelets with a center between 4 Hz and 7 Hz were used in the theta frequency band. At 4 Hz, the temporal precision was 198 ms, and the frequency precision was 0.8 Hz. At 7 Hz, the temporal precision was 113 ms, and the frequency precision was 1.4 Hz. Following convolution in the time domain, the transformed data were averaged across trials, and baseline normalization was implemented using decibel conversion. The following equation was used:

${dB}_{tf}=10log10(\frac{{activity}_{tf}}{{baseline}_{f}})$

where ${activity}_{tf}$ refers to the activity in a frequency of interest at a specific time point, and ${baseline}_{f}$ refers to the mean power of the frequency of interest across the baseline time window.

The decibel conversion was applied in the time window between 700 ms and 200 ms prior to stimulus onset [7]. The data at each time point for a given frequency were normalized to the pre-stimulus power of that frequency on a dB scale. The baseline time window ended prior to the earliest time point that could potentially capture post-stimulus neural activity, considering the temporal resolution based on the lowest frequency analyzed (~200 ms). Consequently, the time window used for baseline correction remained unaffected by post-stimulus activity.

**Supplemental Results**

## *Threat Conditioning (Visit 1)*

### Theta Frequency Band.

###
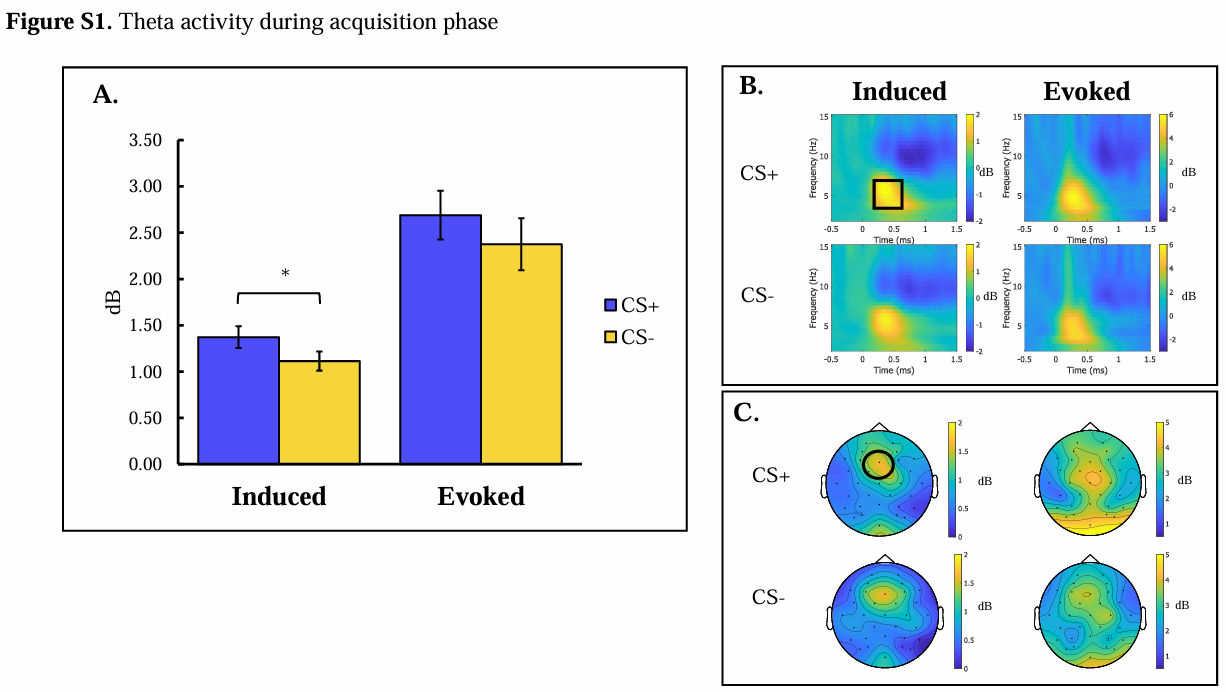
*Induced and Evoked Activity during Acquisition.* See Figure S1

**Figure 1.** Theta frequency band during threat acquisition. **(A)** Mean power of induced and evoked theta activity at frontocentral sites (Fz, FC1, FC2) within 200-600 ms post-stimulus onset. **(B)** Time frequency representation of induced and evoked theta activity at frontocentral sites (Fz, FC1, FC2) during CS+ and CS- trials. **(C)** Topographic presentation of frequency activity 200-600 ms post-stimulus onset during CS+ and CS- trials.

*Notes*: Error bars represent the standard error of the mean; black squares represent the time window used for all statistical analysis; black circles represent the electrodes used for all statistical analyses; CS+, conditioned threat cue; CS-, conditioned safety cue; dB, band-power after decibel conversion.

* *p* < 0.05

***Total Activity.*** RM-ANCOVA with Stimulus (CS+, CS-) x Age (adolescents, adults) x Anxiety yielded a significant main effect of stimulus, *F*(1,96)=6.382, *p*=.013, *η_p_^2^*.062. All participants exhibited greater theta activity for the CS+ (*M*=1.609, *SD*=1.203) than the CS– (*M*=1.347, *SD*=1.044), suggesting successful differentiation in total theta activity. Furthermore, a main effect of age emerged, *F*(1,96)=4.477, *p*=.037, *η_p_^2^*=.045, suggesting adolescents demonstrated overall greater theta activity (*M*=1.704, *SD*=.990) during acquisition than adults (*M*=1.282, *SD*=.990).

### Alpha Frequency Band.

### *Induced and Evoked Activity during Acquisition.* See Figure S2

*
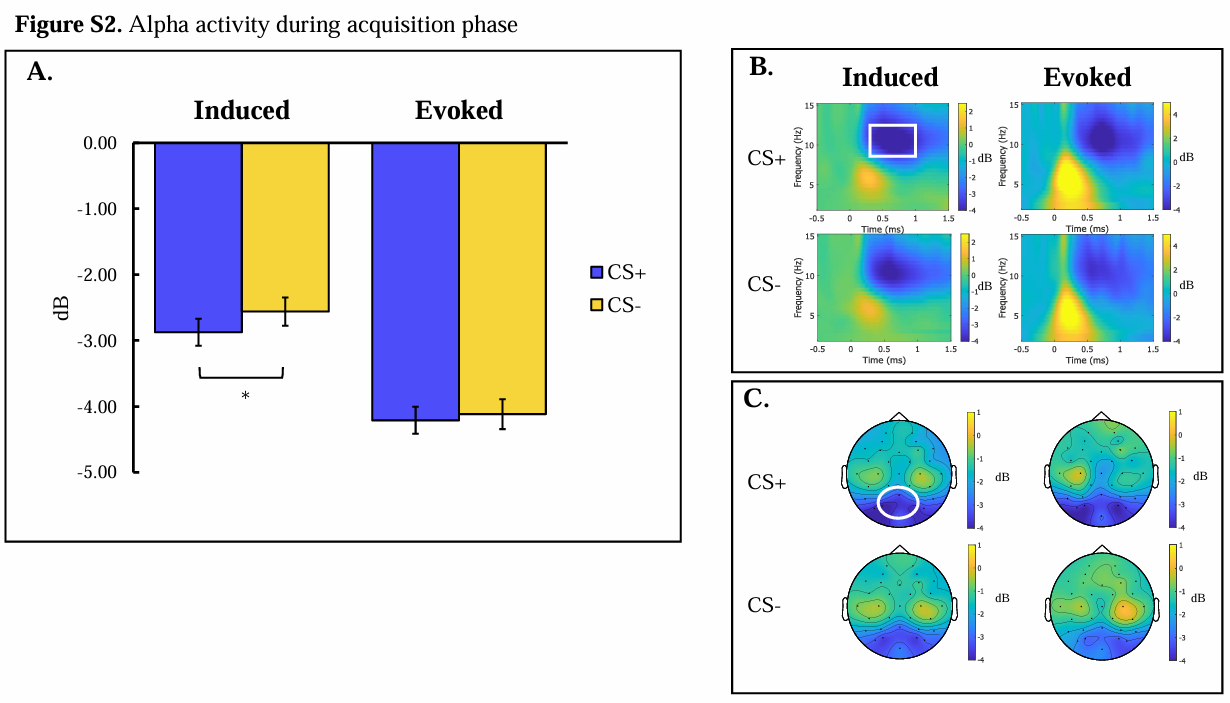
*

**Figure S2.** Alpha frequency band activity during threat acquisition. **(A)** Mean power of induced and evoked alpha activity at occipito-parietal sites (Oz, Pz, PO3, PO4) in a time window of 500-1200 ms post-stimulus onset. **(B)** Time frequency representation of induced and evoked alpha activity at occipito-parietal sites (Oz, Pz, PO3, PO4), during CS+ and CS- trials. **(C)** Topographic presentation of frequency activity 500-1200 ms post-stimulus onset during CS+ and CS- trials.

*Notes*: Error bars represent the standard error of the mean; white squares represent the time window used for all statistical analysis; white circles represent the electrodes used for all statistical analyses; CS+, conditioned threat cue; CS-, conditioned safety cue; dB, band-power after decibel conversion.

* *p* < 0.05

***Total Activity*.** RM-ANCOVA with Stimulus (CS+, CS-) x Age (adolescents, adults) x Anxiety did not yield any significant effects (all *p*s>.093).

## *Threat Extinction (Visit 2)*

### Theta Frequency Band

### *Induced and Evoked Activity during Extinction.* See Figure S3.


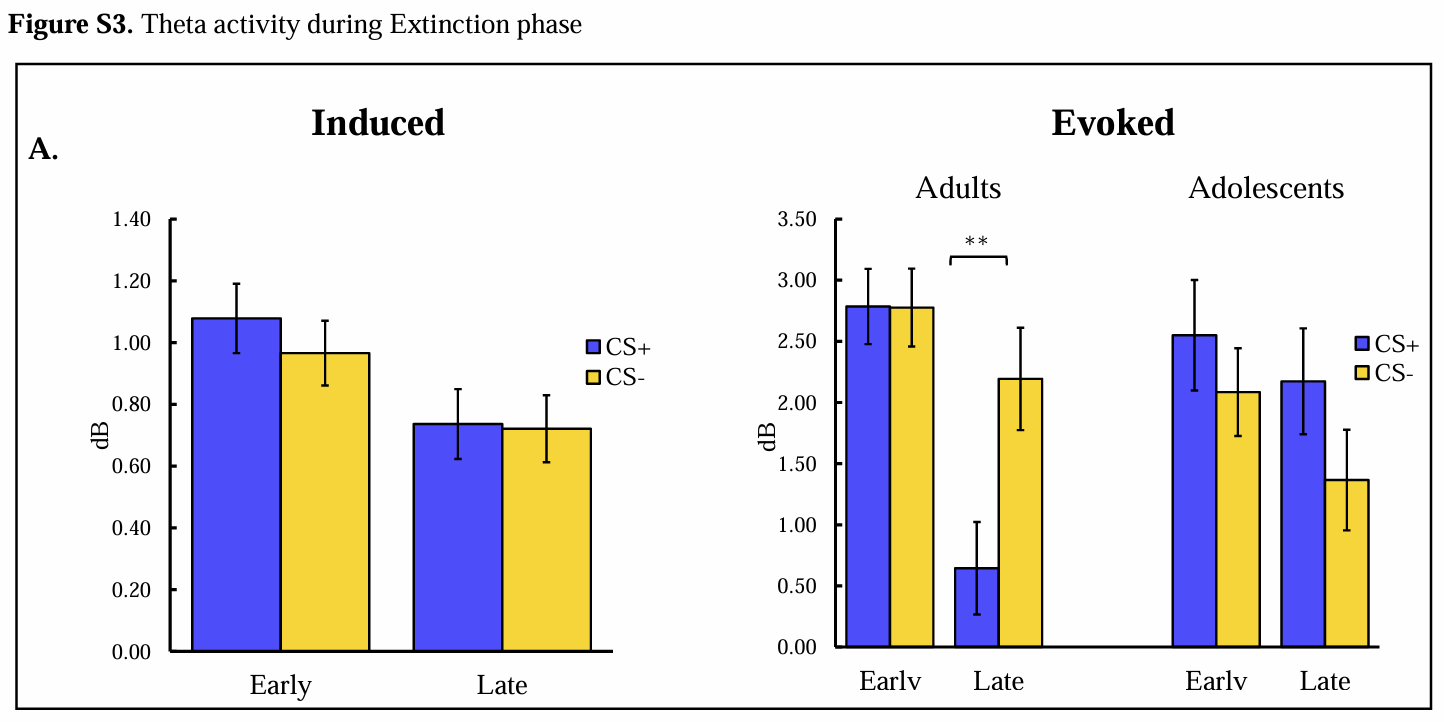

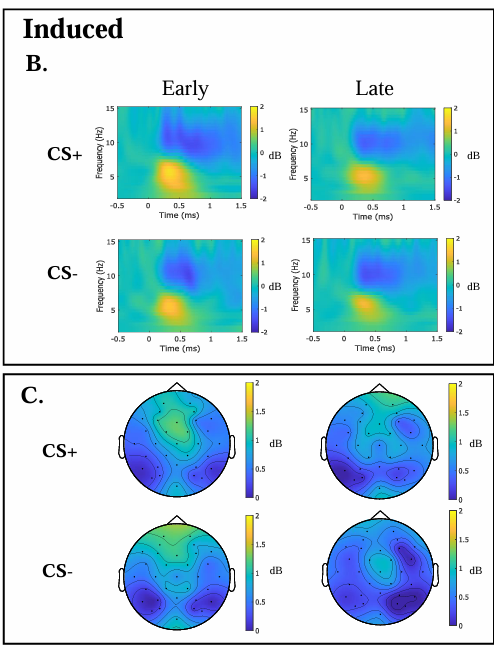

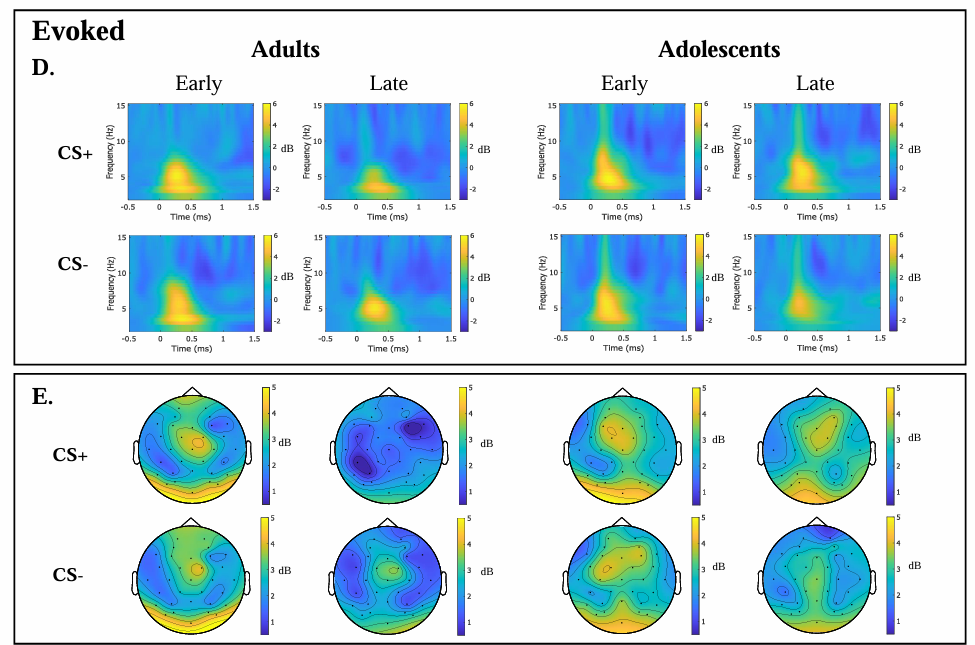


**Figure S3.** Theta frequency band during threat extinction. **(A)** Mean power of induced activity among all participants and evoked theta activity among both adults and adolescents at frontocentral site (Fz, FC1, FC2) in a time window of 200-600 ms post-stimulus onset. **(B)** Time frequency representation of induced activity among all participants at frontocentral sites (Fz, FC1, FC2), during CS+ and CS- trials in early and late phases of extinction. **(C)** Topographic presentation of induced frequency activity among all participants 200-600 ms post-stimulus onset, during CS+ and CS- trials in early and late phases of extinction. **(D)** Time frequency representation of evoked activity among adults and adolescents at frontocentral sites (Fz, FC1, FC2), during CS+ and CS- trials in early and late phases of extinction. **(E)** Topographic presentation of evoked activity among adults and adolescents 200-600 ms post-stimulus onset, during CS+ and CS- trials in early and late phases of extinction

*Notes*: Error bars represent the standard error of the mean; CS+, conditioned threat cue; CS-, conditioned safety cue; dB, band-power after decibel conversion.

* *p* < 0.05; ** *p* < 0.01.

***Total Activity.*** RM-ANCOVA with Stimulus (CS+, CS-) x Phase (early extinction, late extinction) x Age (adolescents, adults) x Anxiety yielded a main effect of phase, *F*(1,90)=9.168, *p*=.003, *η_p_^2^*=.092, suggesting a decrease in overall theta activity during the late phase of extinction.

**Alpha Frequency Band.**

***
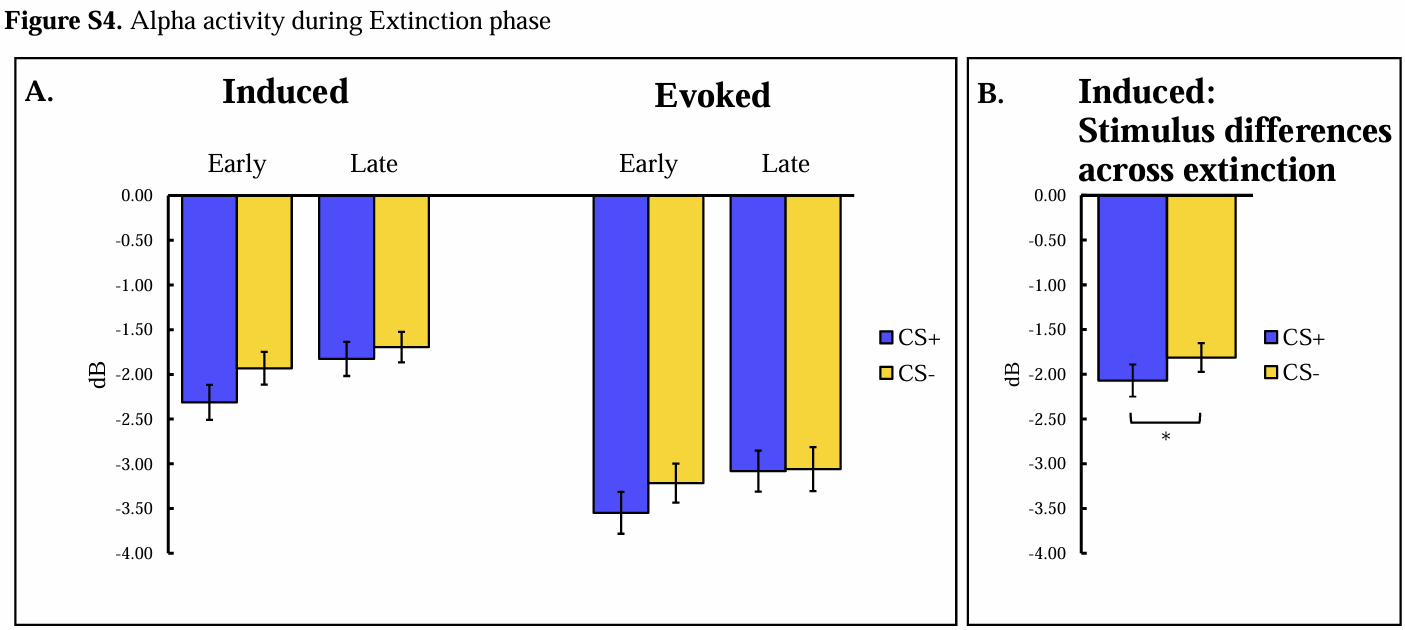
Induced and Evoked Activity during Extinction.*** See Figure S4

**
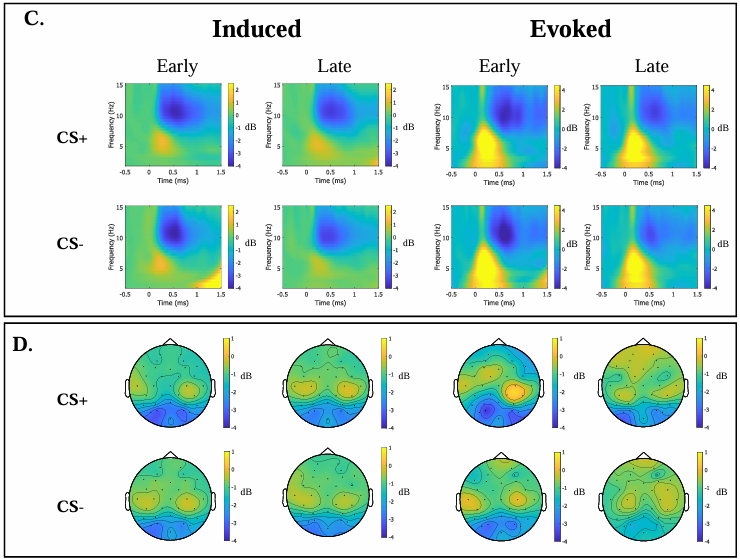
**

**Figure S4.** Alpha frequency band during threat extinction. **(A)** Mean power of induced and evoked activity among all participants at occipito-parietal sites (Oz, Pz, PO3, PO4) in a time window of 500-1200 ms post-stimulus onset. **(B)** Induced activity during CS+ and CS- across all extinction trials. **(C)** Time frequency representation of induced and evoked activity among all participants at occipito-parietal sites (Oz, Pz, PO3, PO4) during CS+ and CS- trials in early and late phases of extinction. **(D)** Topographic presentation of induced and evoked frequency activity among all participants 500-1200 ms post-stimulus onset during CS+ and CS- trials in early and late phases of extinction.

*Notes*: Error bars represent the standard error of the mean; CS+, conditioned threat cue; CS-, conditioned safety cue; dB, band-power after decibel conversion.

***Total Activity*.** RM-ANCOVA with Stimulus (CS+, CS-) x Phase (early extinction, late extinction) x Age (adolescents, adults) x Anxiety yielded a main effect of stimulus, F(1,90)=4.115, p =.045, *η*_p_^2^=.044, suggesting consistent greater alpha suppression (i.e., more negative) during CS+ trials (M=-1.838, SD=1.601) than CS- trials (M=-1.655, SD=1.456). Furthermore, a main effect of phase emerged, F(1,90)=7.598, p=.007, *η*_p_^2^=.078, suggesting a decrease in total alpha activity during the late phase of extinction.

**References**

1. Ishihara S (1917) Tests for color-blindness. Handaya, Tokyo: Hongo Harukicho

2. Cohen MX (2014) Analyzing Neural Time Series Data: Theory and Practice (Issues in Clinical and Cognitive Neuropsychology). MIT Press

3. Debnath R, Buzzell GA, Morales S, et al (2020) The Maryland analysis of developmental EEG (MADE) pipeline. Psychophysiology 57:1–13. https://doi.org/10.1111/psyp.13580

4. Luck SJ (2014) An Introduction to the Event-Related Potential Technique, Second Edi. MIT Press

5. Cohen MX, Donner TH (2013) Midfrontal conflict-related theta-band power reflects neural oscillations that predict behavior. J Neurophysiol 110:2752–2763. https://doi.org/10.1152/jn.00479.2013

6. Shner‐Livne G, Buzzell GA, Fox NA, Shechner T (2023) Induced <scp>error‐related</scp> theta activity, not error‐related negativity, predicts task performance as well as anxiety and worry during <scp>real‐life</scp> stress in a youth sample. Psychophysiology. https://doi.org/10.1111/psyp.14492

7. Bacigalupo F, Luck SJ (2022) Alpha-band EEG suppression as a neural marker of sustained attentional engagement to conditioned threat stimuli. Soc Cogn Affect Neurosci 1–17. https://doi.org/10.1093/scan/nsac029

8. Keil A, Bernat EM, Cohen MX, et al (2022) Recommendations and publication guidelines for studies using frequency domain and time-frequency domain analyses of neural time series. Psychophysiology 59:1–37. https://doi.org/10.1111/psyp.14052
